# Using qualitative evidence on patients’ views to help understand variation in effectiveness of complex interventions: a qualitative comparative analysis

**DOI:** 10.1186/1745-6215-14-179

**Published:** 2013-06-18

**Authors:** Bridget Candy, Michael King, Louise Jones, Sandy Oliver

**Affiliations:** 1 Marie Curie Palliative Care Research Unit, UCL Mental Health Sciences Unit, University College London Medical School, Charles Bell House, 67-73 Riding House Street, London W1W 7EJ, UK; 2 UCL Mental Health Sciences Unit, University College London Medical School, Charles Bell House, 67-73 Riding House Street, London W1W 7EJ, UK; 3 Department of Childhood, Families and Health, Institute of Education, University of London, 20 Bedford Way, London WC1H 0AL, UK

**Keywords:** Complex interventions, Randomised controlled trials, Qualitative evidence, Qualitative comparative analysis

## Abstract

**Background:**

Complex healthcare interventions consist of multiple components which may vary in trials conducted in different populations and contexts. Pooling evidence from trials in a systematic review is challenging because it is unclear which components are needed for effectiveness. The potential is recognised for using recipients’ views to explore why some complex interventions are effective and others are not. Methods to maximise this potential are poorly developed.

**Methods:**

We used a novel approach to explore how patients’ views may explain the disparity in effectiveness of complex interventions. We used qualitative comparative analysis to explore agreement between qualitative syntheses of data on patients’ views and evidence from trialed interventions to increase adherence to treatments. We first populated data matrices to reflect whether the content of each trialed intervention could be matched with suggestions arising from patients’ views. We then used qualitative comparative analysis software to identify, by a process of elimination, the smallest number of configurations (patterns) of components that corresponded with patients’ suggestions and accounted for whether each intervention was effective or ineffective.

**Results:**

We found suggestions by patients were poorly represented in interventions. Qualitative comparative analysis identified particular combinations of components corresponding with patients’ suggestions and with whether an intervention was effective or ineffective. Six patterns were identified for an effective and four for an ineffective intervention. Two types of patterns arose for the effective interventions, one being didactic (providing clear information or instruction) and the other interactive (focusing on personal risk factors).

**Conclusions:**

Our analysis highlights how data on patients’ views has the potential to identify key components across trials of complex interventions or inform the content of new interventions to be trialed.

## Background

Systematic reviews of randomised controlled trial (RCTs) are invaluable in combining evidence on effectiveness. Their interpretation is most straightforward in drug therapies. However, interpretation of the effectiveness of more complex interventions is challenging as the interventions may consist of several components that vary across trials but are assumed to contribute to the effect. Based on reasons of practicality or personal choice policy makers and planners may then select one of these varying interventions. They may also pick certain components contained in one or more of these interventions. New approaches are being sought to increase our understanding of how healthcare interventions exert their effects. These may involve asking trialists to identify the common components of their effective interventions, for example in relation to stroke care units [1]. They may also, based on expert consultation using consensus methods and literature review, involve developing taxonomy to classify and describe effective interventions [2]. Or they may involve seeking to identify mechanisms through which effects of the intervention are achieved. For example, behaviour change theory might be applied to trials identified in a systematic review of ‘audit and feedback’ interventions [3].

Another way is to use recipients’ perspectives, experiences or opinions on the potential suitability and utility of an intervention. Recipients’ views are clearly important, particularly for interventions that require their active participation. Innovative approaches to using such information are being explored, for instance drawing on qualitative evidence on recipients’ (usually patients’) views to inform effectiveness evidence from systematic reviews of trials [4]. The Cochrane Handbook on Systematic Reviews lists various ways qualitative evidence can be used [5]. These include (but are not limited to) helping to define the research question and helping to ensure the review includes important outcomes. Another way is to supplement reviews by synthesising qualitative evidence within a stand-alone, but complementary, quantitative review to address questions on aspects other than effectiveness. In this way it is possible to examine whether interventions that address patients’ priorities might be more effective than those that do not. If this is shown to be the case, it may be possible to identify components that may have more or less influence on outcomes. However, such considerations are constrained by the quality, accuracy and extent of descriptions of the intervention in papers reporting the results of trials [6,7]. Sometimes, there may be no clear description of the intervention beyond a basic statement, for example what telephone counseling or psychosocial care involved [7]. In addition, it remains unclear exactly how best to bring together within a systematic review the different types of evidence (qualitative and quantitative). Moreover, linking qualitative and quantitative evidence may be hampered by the usual custom of publishing different kinds of evidence in separate journals.

In earlier research we used evidence from trials of a complex intervention in which content of the intervention was reported and whose overall effect was unclear [8]. This we combined with evidence from a review of qualitative studies (often referred to as a qualitative evidence synthesis (QES)). We used a data matrix table to align the evidence from the QES of patients’ views with the evidence from a Cochrane systematic review of trials. This allowed visual exploration of correspondence between (i) suggestions on intervention content derived from the QES, and (ii) components of interventions in effective and ineffective trials. We reported that components of effective interventions corresponded more often than those of ineffective interventions with patients’ priorities derived from the QES. The potential of combining mixed evidence in this way is recognised [9].

Qualitative comparative analysis (QCA) is an analytical approach and a set of techniques [10]. It is recognised in social sciences as an innovation in mixed methods research [11,12], and it has been found to be applicable to the evaluation of complex public health interventions [13]. QCA has as yet untested potential for use in integrating reviews of qualitative and quantitative health evidence [14]. The potential of QCA in the analysis of a systematic review of complex interventions is seen in: (1) its usefulness in small datasets (where statistical analysis can be limited), and (2) how it seeks to explain a given outcome by identifying multiple pathways of putative causal factors. This may be viewed as analogous with locating different recipes to make a cake. This approach is relevant to the analysis of complex interventions where the focus should include the differing circumstances, mechanisms and patterns through which an intervention may fit together to exert its effect. In this paper, we report our research using a systematic review with a larger number of quality trials than in our earlier work [8]. This allowed us to test the use of QCA in understanding how to integrate qualitative and quantitative review evidence. In doing so, we aimed to identify more information than would be possible by visual inspection.

In this worked example of combining mixed evidence from systematic reviews on interventions to improve adherence to drug therapy, we used QCA to explore how qualitative evidence might explain the variability in effectiveness of complex interventions.

The objective was to identify matches between patients’ views and components of interventions and see whether these matches were associated with the effectiveness of interventions.

## Methods

### Data

We first identified a systematic review of trials. We searched specifically for a Cochrane systematic review because these reviews are performed to internationally agreed standards. We sought a review on chronic disease self-management because it requires long-term commitment by patients, whose views are therefore pertinent. This review needed to involve a sufficient number of trials to require an analytical tool to aid quantitative analysis (at least five trials) [15]. It also needed to include trials that differed in intervention effect, and in which the content of the intervention was described in sufficient detail to understand how the intervention might be operationalised in practice. We chose a review of interventions to promote adherence to drug therapy for a range of conditions [16], because it fulfilled our criteria and because we were aware from earlier work that a complementary QES was available [8]. This QES by Vervoort and colleagues explored evidence about HIV/AIDS patients’ views on their disease self-management [17]. We purposively sought additional QES for adherence to therapy in other common chronic diseases reported in the trials included in the Cochrane review (such as diabetes, asthma and depression) [16]. We searched three large citation databases from 1999 to 2009; Medline, Psyinfo and Cinahl. We used terms relating to the diseases of participants in the trials and terms describing a QES. Details of the search strings for identifying QES are listed in Additional file 1. As the focus of this study was on the exploratory use of QCA, our search for QES was not exhaustive. We were not concerned that we may not identify a QES in relation to patients’ views on all diseases relevant to the Cochrane review. This is because our earlier research suggested that such evidence was not necessarily disease-specific [8]. We identified three relevant QES [18-20]. They were selected on the basis that (1) they explored patients’ views on living or managing their chronic disease, (2) they were of sufficient methodological quality [21], and (3) their findings were or could be translated into suggestions for strategies to promote adherence to therapy for a range of long-term conditions. The QES by Vervoort et al. identified earlier thematically analysed evidence from 18 studies on patients views on adherence to HIV therapy [17]; of the others, Campbell et al. synthesised evidence in a meta-ethnographic review from 10 studies on patients’ views on diabetes including how they managed their disease [18], the QES by Malpass and colleagues was a meta-ethnography of 16 studies on patients’ experience of taking antidepressants [19] and the other, the QES by Schlomann and colleagues, analysed thematically 11 studies on lay beliefs about high blood pressure [20].

From each QES one author (BC) extracted data on findings relating to promoting adherence. They were listed as individual strategies to promote adherence. The extraction sheet used to extract data from the QES is detailed in Additional file 2. Another author (LJ) checked the list for completeness against the QES. The final list was discussed with all authors at project meetings. From the QES by Vervoort *et al*. we had already derived 22 suggested strategies [17]. The additional three QES provided 17 suggestions; of these, 14 were similar to those generated from the QES by Vervoort *et al*. [17]. They focused on the same issues, for example in the QES on beliefs about high blood pressure, the authors derived as an implication for practice that: ‘The therapeutic content of that consultation is in part dependent on the GP’s understanding of the patients’ beliefs and views regarding medication use’ [20]. This is similar to two suggestions from the QES by Vervoort *et al.*, specifically the more general suggestion ‘Interventionists should enquire into possible factors influencing each individual patient before starting treatment’ and the more focused suggestion advising that ‘ambivalence towards medications should be discussed’ [17]. In total, we listed 25 different ways of promoting adherence. These are listed in Additional file 2: Table S2.

We did not set methodological quality criteria for selection of the review of trials as we assumed a published Cochrane review would have already undergone rigorous checking. However our earlier research suggested the need to restrict trials by quality [8]. Therefore, we only used the 21 trials that were assessed by the Cochrane reviewers as fulfilling their quality criteria [22-42]. This was based on the key recommendation by the Cochrane Collaboration, which was assessment of whether randomization was concealed [43]. The 21 trialed interventions in our chosen Cochrane review varied in how they aimed to promote adherence, for example by providing more instruction, increasing convenience of care, and providing psychological therapy and/or group meetings.

Eleven of the trials were deemed by the Cochrane reviewers as effective in promoting adherence. This judgment was based on whether the *P* value was significant at the <0.05 level. On this basis we assigned a binary outcome for each of the trials; ‘1’ if the trial was effective and ‘0’ if ineffective. We were aware of the weakness of using the *P* value as indicating effect, but additional information was limited. We could not use the preferred option of effect size, because some trials did not report this. Trials also assessed adherence differently, including pill counts, self-report of adherence and pharmacist records. The data extraction form for whether the intervention was found to be effective is provided in Additional file 2: Table S3.

#### Conjoining extracted data

We scored the contents of each intervention against the 25 suggested strategies derived from the QES. The information on intervention content was key to our analysis. Details of the interventions were sought from the original trial papers in which they were more extensively reported. However, to ensure detail was sufficient for our analysis, on reading the papers we applied two criteria: (1) that more than two sentences were devoted to describing the intervention content, and (2) that components were described in detail [44]. What we meant by detail was that there was more than a brief statement. For instance, if the intervention was described as educational, then there needed to be detail on more than one aspect of that educational approach. All intervention descriptions met these criteria. Intervention descriptions ranged in length from three sentences to twenty-eight (the median in interventions found ineffective was six to eight, and in effective was nine to ten). The description for each trial intervention is provided in Additional file 3. We used binary scoring; 1 = suggestion corresponded with an intervention component, 0 = no correspondence. We were aware that binary scoring could reduce the potential information available. However, a more graded scoring was for many suggestions not relevant, and if adopted might have increased subjectivity in scoring. To enhance standardisation and clarity, scoring guidelines (available from authors) were devised and tested by independent researchers. We populated a data matrix table for each intervention with all the conjoined data and where the intervention was effective. We report an analysis of the relationships created in this table between the qualitative and quantitative data.

#### Qualitative comparative analysis: the approach

To analyse the relationships created we used QCA [10]. QCA uses Boolean algebra. It is grounded in set-theoretic relationships. This allows in the analysis all logically possible combinations of factors to be examined systematically in relation to the outcome: as in which ingredients work together to make a cake. In our worked example, QCA identifies across interventions all parsimonious patterns of components that match patients’ views and result in the intervention being effective. Here, parsimonious is the smallest number of patterns of components (in the dataset) that account for the intervention effect. This is not to imply that an intervention is effective because of the match with patients’ views but rather this congruence might moderate its effectiveness.

QCA is case-orientated in that it seeks to explain the outcome in terms of ‘pathways of components’ per trialed intervention: as in which ingredients per cake were essential or necessary. This differs from a conventional statistical approach, which would average the effect simultaneously across all interventions. Therefore, in this example QCA presents ‘key’ components per individual intervention. ‘Key’ in these data means those found to be the simplest explanation (in the data provided) for the results.

#### Qualitative comparative analysis: the technique

To explore patterns in the relationships between the data we used specific QCA analysis software [45]. The software explored these data using Boolean algorithms. This involved pair-wise comparison between interventions to establish commonalities in the way the relationships created (between the qualitative and quantitative evidence) combined together in effective and ineffective interventions. To do this the QCA software reduced the data presented per intervention by only retaining those data found to produce the outcome.

**Figure 1:**
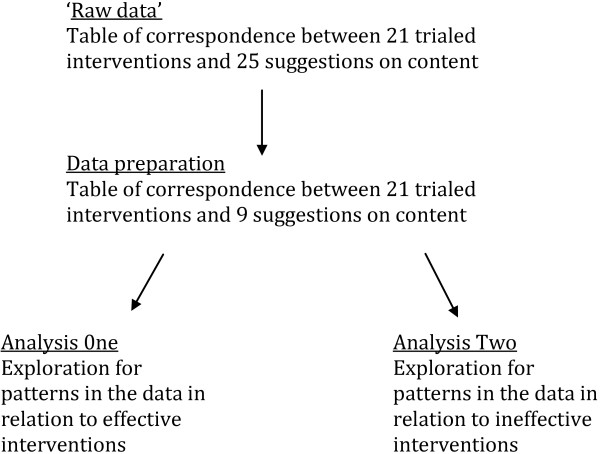
Flow chart for QCA analysis.

The analytical process has several stages; these are charted in Figure 1. QCA involves analysis of cases, factors and outcomes. In these data cases are the interventions and outcome is whether or not the intervention under trial conditions was found to be effective. Factors were the relationships created between the suggested strategies and the intervention content, that is whether or not the intervention contained content that corresponded with patients’ views on how to promote adherence. QCA is limited in the number of factors that can be included in analysis. This is because cases must be aligned with factors and this process can quickly become unwieldy. This is due to the large number of possible combinations of factors that are created. For two factors there will be four possible configurations, for nine factors there will be 512 and so on. Thus the number of possible configurations of factors can quickly exceed the number of cases. On examining published papers using QCA, we found that none used more than 10 factors. In our worked example, we reduced our number of suggested strategies from 25 by first not including those that had been taken up by three or fewer of the trialed interventions. We then combined those that had some overlap in aim. This resulted in nine suggested strategies. See Table 1.

**Table 1:** Reducing for the purposes of qualitative comparative analysis (QCA) the number of factors to below 10

***First step. Excluding 10 factors that had been described in three or fewer interventions:***	1. Acknowledging within the intervention that adherence is dynamic.
	2. Paying attention to possible negative social circumstances.
	3. Discussing whether secrecy of disclosing condition is threatened by taking treatment.
	4. Discussing the seriousness of the disease.
	5. Feedback about positive reactions of the body to treatment should be provided.
	6. In cases of depression, this should be treated before starting therapy; substance misuse should be managed as a first priority.
	7. To develop a trusting relationship with the patient.
	8. To facilitate to learn to trust in oneself.
	9. To get patients to describe their own behaviour.
	10. To offer good medical follow-up.
***Second step. Combining factors that had some similarity in aim:***	11. Enquire into personal risks factors, and 12. Use insight on personal risk factors became ‘a focus on personal risk factors’;
	13. Discuss ambivalence to medicine, and 14. Discuss acceptance of disease became ‘an exploration of attitudes to drug and/or disease’;
	15. Pointing out the value of treatment to a patient’s life enhances motivation, and 16. Explain the relationship between adherence and disease became ‘emphasis on the value of adherence’;
	17. Clear instructions on how to take medication, and 18. Information appropriate to patient’s understanding became ‘clear or appropriate information’;
	19. Acquire insight into a patient’s social support systems, 20. Counsel patient on how use social support, and 21. Social support has to be substantial and practical became ‘a focus on improving social support’.
***Final list of factors used in QCA (this is the four remaining factors (22 to 25) and the five combined factors (a to e)***	22. Discuss circumstances that lead to forgetting to take treatment.
	23. Emphasise that experiencing no symptoms does not mean to stop taking the drug.
	24. Enhance convenience of taking the drug.
	25. Information on side effects.
	a. A focus on personal risk factors.
	b. An exploration of attitudes to drug and/or disease.
	c. Emphasis on the value of adherence.
	d. Clear or appropriate information.
	e. A focus on improving social support.

QCA analysis is unidirectional; therefore we undertook two analyses, one for effective and a second for ineffective interventions.

### Analysis models

QCA uses three analysis models to summarise findings [10]. These are the complex, intermediate and the parsimonious. We present the parsimonious model because, unlike the complex model, it fully utilises the mathematical approach by allowing inferences to be made on unobserved cases. Inferences are based on the patterns found between the data in the observed cases; the model makes assumptions on the outcome of the combinations of factors not taken up in the unobserved cases. In the complex model, no inferences are made on unobserved cases. In a sensitivity analysis, we also ran the analysis using this model. In the intermediate model, the researcher makes assumptions on unobserved cases by testing the effect in one selected direction only (as in a one-tailed statistical test). We did not use this model as a suggested strategy could have potentially led to a negative outcome.

### Sensitivity analysis

Our approach involved subjective decisions that could impact on our findings. Therefore, we tested the underlying assumptions in our model by:

• reducing data by (1) excluding cases (interventions) if they did not correspond with any of the suggestions (factors); and (2) removing certain suggestions because they did not enter the final analysis model or were found in analysis to explain the outcome by both their presence or their absence;

• increasing the data by including all 67 trialed interventions (irrespective of quality) in the Cochrane review;

• analysing the data differently by (1) reporting the results using the QCA complex model; and (2) using (where appropriate) a graded scoring system rather than binary to score agreement between intervention components and suggestions.

## Results

We found that suggestions by patients were poorly represented in interventions. In Additional file 4 this is illustrated in a cell by a ‘–’. QCA identified six potential pathways (configurations of components) to an effective and four to an ineffective intervention. The configurations are different per outcome. Each configuration is represented in Additional file 4 by a different colour shading, border or cell pattern.

### Effective interventions

There were two types of patterns for the effective interventions; one involving the presence of one component (pathway 1), and the others involving more components of which some are shared between the configurations (pathways 2 to 6).

The configuration most commonly associated with effectiveness (found in eight trials) involved one component: ‘a focus on personal risk factors’ (pathway 1: [22-24,26,27,30,33,34]). Other configurations each involved either the presence of ‘explaining the value of adherence’ or ‘provision of clear/appropriate information on how to take medication’, and the absence of other components (Table 2 and Additional file 4: Table S1).

**Table 2:** Pathways identified to effective and ineffective interventions

***Effective interventions***	The configuration most commonly associated with effectiveness (found in eight trials) involved one factor: ‘a focus on personal risk factors’ (pathway 1: [15-17,19,20,23,26,27]). Other configurations each involved the presence of one factor, and the absence of other factors. One configuration involved ‘explaining the value of adherence’ with the absence of:
	(i) ‘Discuss circumstances that lead to forgetting to take treatment’ and ‘a focus on improving social support’ (pathway 2: [24,25,29]).
	Or the absence of:
	(i) ‘Discussion relating to not stopping the medication if there are no symptoms’ and ‘improving social support’ (pathway 3: [16,18,22,29]).
	The other configurations involved ‘provision of clear/appropriate information on how to take medication’, with the absence of:
	(i) ‘Exploration of attitudes to therapy/disease’ and ‘discussion relating to not stopping taking medication if there are no symptoms’ (pathway 4:[17,18,22,29]).
	Or the absence of:
	(ii) ‘Discussion relating to missing a drug’ and ‘discussion relating to not stopping taking medication if there are no symptoms’ (pathway 5: [24,25,27,29]).
	Or the absence of:
	(iii) ‘Discussion relating to not stopping taking medication if there are no symptoms’ and ‘improving social support’ (pathway 6: [17,18,22,29]).
***Ineffective interventions***	All four configurations (pathways) for the ineffective interventions included the absence of one factor: ‘a focus on personal risk factors’. Two of the configurations also involved the absence of either:
	(i) ‘Information on side effects’ and ‘pointing out the value of adherence’ (pathway 1: [31,33-35]).
	or
	(ii) ‘Pointing out the value of adherence’ and ‘provision of clear or appropriate information’ (pathway 2: [30,33-35]).
	In the other two configurations the absence of ‘a focus on personal risk factors’ also involved the presence of either:
	(i) ‘Discussion relating to missing a drug’ (pathway 3: [28,30-32]). or
	(ii) ‘Emphasis that experiencing no symptoms does not mean stopping medication’ (pathway 4: [21,32]).

The pathways correspond with two distinct approaches to promoting adherence, one being interactive (‘a focus on personal risk factors’) (pathway 1) and the other more didactic (‘emphasizing clear or appropriate information’) (pathways 2 to 6). Neither the interactive nor the didactic approach was differently associated with the specific diseases in the trial populations.

### Ineffective interventions

All four configurations (pathways) for the ineffective interventions included the absence of one component, ‘a focus on personal risk factors’. The configurations also involved various combinations of other components (Table 2 and Additional file 4: Table S2).

### Sensitivity analyses

There were sufficient data to undertake all proposed sensitivity analyses. None of the analyses had dramatically different findings, in that most of the dominant patterns per outcome remained. A common component, ‘a focus on personal risk factors’, for an effective intervention remained in most analyses. As before, the configurations for ineffective interventions commonly remained the mirror opposites of those found in the models for an effective outcome. As expected, the sensitivity analyses produced more configurations. Some of these (particularly when all trials irrespective of quality were included) were conflicting, in that they included per outcome both the presence and absence of certain factors.

## Discussion

### Main findings

We tested QCA as a way to combine QES evidence on patients’ views with evidence from a systematic review of trials of a complex intervention. We used as a worked case example evidence on interventions to promote adherence to long-term drug therapy. We found in general, patients’ suggestions on what is important were poorly represented in the interventions. Using QCA we found that certain suggestions seemed to hold together in particular patterns that corresponded to whether the intervention was effective or not. Three influential components were identified in the effective interventions, while other components were influential through their absence. It seemed that those that were influential by their absence were those in which those providing the intervention focused on negative influences on adherence, such as ‘the need to take the drug continuously irrespective of symptoms’. The pattern of components suggests two approaches to promoting adherence. One involved adopting an interactive style focusing on personal risk factors. The other was a more didactic approach. This involved emphasising clear, appropriate information including the value of adherence but without discussing certain potentially challenging aspects, such as discussion of attitudes to medication or disease, missing doses or enhancing social support, or taking the drug continuously irrespective of symptoms. Individual patients may respond better to either the interactive or didactic approach.

It is difficult to identify other research that has similar overall findings, in part because of the novelty of this approach. However, what we found had face validity. In particular, the correspondence between effective and ineffective interventions was often mirror opposites. This was best illustrated by the suggestion that the intervention should focus on personal risk factors, which was present in most of the effective interventions and absent in all ineffective interventions. These findings generate hypotheses about what may be more usefully included in future interventions, which then need to be followed by randomised controlled trials to generate evidence about effectiveness.

### Limitations and advantages

There are challenges to the use of QCA as used in this study in the evaluation of interventions. These make interpretation of our findings more difficult. First, prior to QCA analysis, selection and processing of the evidence from source to our analysis involved the need for several decisions. As the focus of this study was on the exploratory use of QCA, our search for QES was not exhaustive. We used only controlled vocabulary and a limited number of databases. This presupposes that there are more QES beyond those located, from any further QES there may be other ways to promote adherence. There were several stages of synthesis, extraction and interpretation, with only the final stages undertaken by us. For the qualitative evidence this involved: (1) patients’ views on living with and managing a disease being collected and analysed in a large number of studies; (2) the findings from these studies being pooled in further qualitative analysis, which used different methodologies in critiquing and analysing; and (3) these findings then being translated into suggestions for intervention components. In the quantitative evidence, the method was challenged by reliance on the completeness of intervention descriptions. Complex interventions are often poorly described in main trial papers and this is a real limitation to any attempt (using QCA or another method) to understand why variations in effectiveness occur. The brevity of descriptions of the intervention and the lack of agreement over what constitutes a complete description make it problematic to assume that particular aspects of an intervention were absent simply because they were unreported. Another limitation in our work is using a *P* value to decide whether or not an intervention was effective or not. However, wherever possible, we used robust approaches to reduce the risk of bias, such as by setting quality criteria, and making independent checks of steps undertaken during the study.

In our use of QCA we also needed to make subjective decisions that could have introduced error. However, we undertook sensitivity analyses to test the consistency of our findings. In QCA a limited number of factors can be analysed and this restricted our exploration of heterogeneity of interventions to less than ten features. Additionally, within any one (QCA) analysis, the exploration of an outcome can only be conducted in one direction (either the outcome is effective or ineffective). A single analysis cannot be used to quantify and compare head to head the differences between the outcomes. The method also does not incorporate a statistical (probability) measure of the precision of the relationships found in the data, or the likelihood that they are simply chance findings. However, the small dataset limits the use of conventional statistics and thus alternative approaches are needed. Finally, QCA is only of use in circumstances where the trial evidence continues to result in equivocal results. Thus not all interventions have the potential to undergo this sort of analysis.

This case study illustrates the potential for QCA to address a key challenge in trials of complex interventions, namely understanding why some are effective and others not. It is particularly useful where the pathways (as in complex interventions) to success may differ across trials. Using QCA to analyse complex healthcare interventions could identify several types of best practice; that is, effective interventions that share similar *key* components. Thereby planners and practitioners may be informed by patient choice to select intervention components for a type of best practice based on local conditions and choice. This is with an understanding of which components it may be more important to include.

QCA is under-explored and its usefulness in complex intervention development is not established. This study does not suggest that this approach may be useful in every circumstance, or that QCA is more informative than other approaches being considered. Moreover, there is value in consulting patients directly in the development of an intervention [46]. While the results of local consultation apply to the specific setting, they also shed light on implications of the intervention elsewhere and the need for consideration of recipients’ views and behaviours. For instance, Atkins and colleagues [47] explored patients’ experiences of a new intervention, aiming to empower them to take more responsibility for the management of their tuberculosis. They found that the intervention had achieved its aims but only in patients internalising the intervention messages, not necessarily resulting in an increase adherence.

This paper highlights the value of listening to patients’ views in order to understand disease management. In the case of chronic disease, greater adherence seems to be associated with a focus on personal risk factors, an emphasis on the value of adherence, and the provision of clear and appropriate information on how to adhere. We have demonstrated the potential value of using qualitative research to explain the varying effectiveness of complex interventions. We call for more integrative research in this area.

The usefulness of QCA should be tested by comparing it with alternative approaches such as using more subjective researcher judgments in exploring the same evidence, or (dependent on data limitations) with other analytical tools such as such as Bayesian statistics or regression methods. The 10 factors (listed in Table 1) that were mentioned in three or fewer interventions and omitted from the analysis may deserve further attention from primary research.

## Conclusion

In this case study, we found that the application of QCA enhanced our understanding of the effectiveness of complex interventions.

## Abbreviations

QCA: Qualitative comparative analysis; QES: Qualitative evidence synthesis; RCT: Randomised controlled trials.

## Competing interests

The authors declare that they have no competing interests.

## Authors’ contributions

BC contributed to the conception and design of the project, the analysis and interpretation of data and to drafting the manuscript. LJ contributed to the design of the project, the interpretation of data and to critically revising the manuscript for important intellectual content. MK contributed to the conception and design of the project, the interpretation of data and to critically revising the manuscript for important intellectual content. SO contributed to the conception and design of the project, the interpretation of data and to critically revising the manuscript for important intellectual content. All authors read and approved the final manuscript.

## Supplementary Material

Additional file 1 Search strings for identifying qualitative evidence synthesis. Click here for file

Additional file 2: Table S1 Data extraction from the qualitative evidence synthesis with example in italic. **Table S2.** Data extraction from the intervention descriptions and twenty-five ways of promoting adherence. **Table S3.** Data extraction from the Cochrane review with example in italic. Click here for file

Additional file 3 Intervention descriptions as reported in trial papers. Click here for file

Additional file 4: Table S1 Representation of suggestions in effective trials and QCA solution displaying the six pathways to effectiveness. **Table S2.** Representation of suggestions in ineffective trials and QCA solution displaying the four pathways to ineffectiveness. Click here for file
