# Supplementary material for: Using qualitative evidence on patients’ views to help understand variation in effectiveness of complex interventions: a qualitative comparative analysis
Source: Trials. 2013 Jun 18;14:179. doi: 10.1186/1745-6215-14-179 (PMC3693880; doi:10.1186/1745-6215-14-179)
Supplement: Additional file 1 — Search strings for identifying qualitative evidence synthesis. [file 1745-6215-14-179-S1.doc]

Additional file 1: Search strings for identifying qualitative evidence synthesis

1. qualitative synthesis or qualitative review or qualitative evidence synthesis or meta-ethnography or interpretive synthesis

2. diabetes or asthma or depression or lung disease or hypertension or schizophrenia or heart disease

3. (#1 OR #2)
