# Supplementary material for: Using qualitative evidence on patients’ views to help understand variation in effectiveness of complex interventions: a qualitative comparative analysis
Source: Trials. 2013 Jun 18;14:179. doi: 10.1186/1745-6215-14-179 (PMC3693880; doi:10.1186/1745-6215-14-179)
Supplement: Additional file 2: Table S1 — Data extraction from the qualitative evidence synthesis with example in italic. Table S2. Data extraction from the intervention descriptions and twenty-five ways of promoting adherence. Table S3. Data extraction from the Cochrane review with example in italic. [file 1745-6215-14-179-S2.doc]

**Additional file 2: What data was extracted from each source (Tables 1,2, and 3) and twenty-five ways of promoting adherence (Table 2)**

**Table S1: Data extraction from the qualitative evidence synthesis with example in italic**

| Reference for QES | Review findings in relation to promoting adherence |
| --- | --- |
| *Example: Campbell 2003 ** | ** Time has to pass and experience of monitoring and observing one’s body and its reactions has to be gained.*  ** Trust in one’s own actions and observations have to be acquired.*  ** A less subservient/more questioning approach to- wards care providers has to be fostered.*  ** Some knowledge of the mechanisms of diabetes has to be acquired.*  ** The seriousness of diabetes has to be acknowledged.*  ** The person with diabetes needs supportive care providers who supply information, help with monitoring and attempt to understand the person’s self-care strategies rather than judging them.* |

* How the final list of suggestions on promoting adherence was derived is detail in the paper.

**Table S2: Data extraction from the intervention descriptions and twenty-five ways of promoting adherence**

| **Listed below are the 25 suggestions derived from the QES. Any correspondence between trial intervention descriptions was listed below a suggestion. See example in italic.** |
| --- |
| 1: Acknowledgement that adherence may vary over time. Therefore ongoing attention to adherence should have highest priority.  2: Interventionists should acquire into possible factors influencing each individual patient before starting treatment.  *For example: ‘Each experimental patient was interviewed to identify any daily habits or rituals’ Haynes 1976.*  3: Interventionists should use insight into possible factors influencing each individual patient before starting the treatment.  4: Medication should be adapted to patient’s life rather than life to medication: e.g. use of a watch or pill-box with an alarm to remind a patient to take medication – (unwanted) disclosure can be avoided.  5: Clear information should be give on side effects and how to manage them, including those that may be unpleasant and distressing, to ensure maximum understanding.  6: During every visit any ambivalence towards medications should be discussed.  7: Patients’ acceptance of their disease status should be discussed bearing in mind that taking medication can renew confrontation with diagnosis on a regular basis.  8: Secrecy is threatened by taking treatment – the possibility of disclosure should be discussed as openness leads to higher adherence: if disclosure is not an option, the patient can be devised how to take medication in secret to avoid skipping doses.  9: The interventionist needs to highlight the seriousness of the condition to the patient.  10. Feedback about positive reactions of the body to treatment should be used at each visit to support adherence e.g. decreased viral load.  11 Pointing out the value of treatment to a patient’s life enhances motivation.  12. Information transfer appropriate to a patient’s level of understanding will lead to the patient having correct knowledge of what constitutes good adherence practice.  13. It is important to get patients to describe their own behaviour so as to pick up any risky patterns and incorporate them into approach to ensuring adherence.  14. Regular discussion of the details of circumstances that lead to forgetting medication can reveal aspects that need attention to improve adherence e.g. capacity to organise life and activities and to anticipate risky situations.  15: In case of depression, this should be treated before starting therapy; substance misuse should be managed as first priority.  16: To facilitate the learning to trust in ones own actions and observations.  17: To develop a trusting/positive/supportive relationship/partnership with the interventionist.  18: To give clear instructions on how to take medication.  19: Explain the relationship between adherence and disease.  20: Offer good medical follow up.  21: Emphasis that disease may or may not be with symptoms at any time and this does not mean that absence should be used as an indication to stop medication.  22: Acquire insight into a patient’s social support systems.  23: Counsel on how to use their social support systems.  24: Attention to possible negative social circumstances e.g. mothers of young children may need help to fit medication into hectic schedules.  25: Social support has to be substantial and practical – such as reminders. |

**Table S3: Data extraction from the Cochrane review with example in italic**

| Reference for trial | Was the intervention found to be effective in increasing adherence*? |
| --- | --- |
| *Example: Haynes 1976* | *Yes* |

*This was based on significant p-value at >0.05 (Discussion of the limitations of this are documented in the paper).
