# Supplementary material for: Using qualitative evidence on patients’ views to help understand variation in effectiveness of complex interventions: a qualitative comparative analysis
Source: Trials. 2013 Jun 18;14:179. doi: 10.1186/1745-6215-14-179 (PMC3693880; doi:10.1186/1745-6215-14-179)
Supplement: Additional file 3 — Intervention descriptions as reported in trial papers. [file 1745-6215-14-179-S3.doc]

**Additional file 3: Intervention descriptions as reported in trial papers**

**Berrien 2004**

The intervention consisted of eight structured home visits over a 3-month period by the same home care experienced registered nurse (ER). The visits were designed to improve knowledge and understanding of HIV infection, to identify and resolve real and potential barriers to medication adherence, and ultimately to improve adherence. Knowledge questions were designed to measure the subjects’ and/or caretakers’ basic understanding of HIV infection, whether or not they could name the medications and the proper dose, how the medications work, and the potential difficulties that result from not taking medications or missing doses. These questions also related to the caretakers’ pharmacy skills (reading the label, need for monthly refills, and interacting with the pharmacist) and their ability to inform the physician or clinic nurse about medication problems. In the familiar surroundings of home, the research nurse reviewed the pre-intervention questionnaire results with the patient and caretaker. In this first visit, the nurse established a therapeutic relationship with the family. Together they determined the specific problems that existed with respect to knowledge and understanding of HIV infection, and current antiretroviral medication adherence. During this visit, she emphasized the importance of medication adherence in controlling the symptoms of HIV disease. A Spanish-speaking case manager was present if translation was needed. Successive home visits continued to emphasize HIV education and medication adherence. The patient or caretakers were asked to record in a home notebook the individualized plan of care and the progress made toward overcoming specific problems relating to medication adherence. The parent or caretaker and the child participated in the learning process in their own ways, filling out notebooks with stickers for completed medication doses and receiving prizes from the nurse’s “bag of tricks.” These incentives included medication boxes, pill cutters, pill crushers and beepers as well as small toys, diaries, and age-appropriate items. Children and parents learned how these devices could help with adherence. They also learned to name the medications, the dosing, and the frequency of refilling, and how to work comfortably with the pharmacist and clinic nurse. Families were helped to overcome identified barriers to medication adherence during the course of the eight visits. A comic book, “B- Cell, T-Cell Tales,”22 and a video, “Kids-to- Kids: Medication Dedication,”23also were used to teach about HIV infection in those families where the children knew their HIV diagnosis. Alternatively, the parents could view these separately.

Education eventually involved a role reversal in which the nurse became the student with parent and child teaching what they had learned. In another attempt to enhance medication adherence and options for treatment, the nurse offered pill-swallowing training. During the last home visit, the nurse and family assessed the progress they had made in achieving their goals toward knowledge and understanding of HIV infection and in adhering to the medication regimen as prescribed.

The research nurse then administered the same multiple-choice questionnaire, now as a post- study test. Of note, the educational intervention was directed to the patient and caretaker equally, but in a manner suitable to the HIV disclosure status of the child.

**Farber 2004**

Children (with their accompanying adult) who were randomized to the intervention group received an asthma education and management intervention, described in Table 2.The intervention was conducted either as part of the ED visit or as part of the hospital stay (if the patient was admitted to the hospital from the ED). The intervention was implemented by research staff (H.F., as pediatric pulmonary fellow; or L.O., as research nurse; or both).

Subjects in the intervention group received basic asthma education; instruction on use of a metered-dose inhaler with holding chamber; a written asthma self-management plan illustrated by zones colored green, yellow, and red; a sample age-appropriate holding chamber; and prescriptions for medication needed to implement the plan. This medication included an inhaled corticosteroid drug for everyday use and a quick-acting bronchodilator for use as needed. The importance of seeking urgent medical care in the red zone was emphasized. Written asthma plans were consistent with those recommended in the (then-current) 1991 National Asthma Education and Prevention Program Expert Panel Report.The plans used text, peak flow measurements, and pictures to describe the green, yellow, and red zones15and used stickers with pictures of medication to help subjects identify the medication. The intervention was conducted by the investigators (H.F., L.O., or both).

Three brief follow up phone calls were placed to patients in the intervention group at 1–2 weeks, 4–6 weeks, and 3 months after enrollment. The goal of the telephone calls was to reinforce asthma management skills, including use of the green/yellow/red zone plan and adherence to use of daily inhaled anti-inflammatory medication. Return to a pediatrician or asthma specialist was suggested when asthma control was poor.

TABLE2. ASTHMA EDUCATION AND MANAGEMENT INTERVENTION

1. Show brief video (Kids and Asthma).

2. Discuss goals, fears, and concerns.

3. Describe normal lungs, use Open Airways for Schools poster 1.

4. Describe what happens to the lungs during an asthma episode, use Open Airways for Schools poster 2.

5. Describe role of preventive (controller) versus symptom-relieving medication.

6. Describe principles of an asthma management plan illustrated by zones colored green, yellow, and red. Use Open Airways for Schools poster 3.

7. Write out Asthma Self-Management Plan illustrated by zones colored green, yellow, and red. Use self- management plan with pictures describing severity zones.15Use stickers with pictures of inhalers to identify medication. Obtain approval of plan and necessary prescriptions from the patient’s treating physician. Medication treatment plans were consistent with National Asthma Education and Prevention Program Expert Panel Report recommendations.

8. Discuss asthma triggers. Use Open Airways for Schools poster 11.

9. Reinforce asthma management behavior. Use Open Airways for Schoolsposter 4 (Know Your Warning Signs), poster 6 (Take Your Asthma Medicine), poster 7 (Rest and Relax), poster 10 (Red Light, Yellow Light, Green Light), and poster 13 (Instead of Worrying, What Can You Do?).

10. Teach inhaler/holding chamber technique. Give subject sample inhaler and holding chamber. Demonstrate correct use. Have patient give return demonstration.

**Haynes 1976**

The experimental group received the following set of strategies:

Home Self-measurement of Blood-pressure

Each experimental patient was loaned an aneroid sphygmomanometer and stethoscope and instructed in its use. 18 men were loaned separate cuffs (’Nelkin’ sphygmomanometer model 204M; Nelkin Medical Products, Inc., Kansas City, Missouri) and stethoscopes; two men with upper extremity impairments were loaned devices in which the stethoscope head was incorporated into the cuff (’Arden’ sphygmomanometer- stethoscope model HRI 8104-705201; Taylor Consumer Products Division, Sybron Corporation, Arden, North Carolina).

Home Blood-pressure and Medication Charting.

Each experimental patient was issued with daily pill and blood-pressure charts and asked to record (by number and by a dot on the chart) his fifth-phase blood-pressure each day, along with both the number of pills taken and the number of pills missed during the previous day. The treatment goal of a fifth- phase blood-pressure below 90 mm Hg was clearly stated, and the background of the blood-pressure chart was red above, and, blue below, this value.

Tailoring

Each experimental patient was interviewed to identify any daily habits or rituals. The resulting pattern was compared with the patient’s antihypertensive regimen and when the two coincided, agreement was sought to take the pills immediately before executing the habit or ritual; it was also suggested that medications be placed at the sites of these rituals.

Increased Supervision and Reinforcement

Experimental patients were asked to report fortnightly for a review of their daily pill and blood-pressure charts and for a check of their blood-pressure. At each review, if the blood- pressure check was either < 90 mm Hg or > 4 mm Hg below that observed at the sixth month, the patient was praised and received a$4 credit toward ownership of the home blood-pressure cuff and stethoscope. Praise was also received for periods of perfect compliance, and the reasons for every missed pill were sought in an effort to identify problems and solve them through further tailoring. If neither a blood-pressure fall nor perfect compliance had occurred, the patient was encouraged to do better over the next interval.

**Hill 2001**

The intervention group received a comprehensive programme of patient education based on the theory of self efficacy: a person’s conﬁdence in their ability to perform a speciﬁc task or achieve a certain objective. Patients who exhibit a high degree of self efficacy believe that they can make a positive difference to their own health. The programme comprised information about the types of drugs used for rheumatoid arthritis, the disease process, physical exercise, joint protection, pain control, and coping strategies. Written information, including a drug information leaﬂet developed specially for the study, was provided as back up. The non-education cohort received the same drug information leaﬂet as the intervention group. This was in question and answer format and supplied information about the drug, how and when to take it, unwanted side effects, and described safety monitoring. To ensure readability it was written at an easy to read level as measured by the Flesch Reading Ease Index.

**Kemp 1996**

The intervention consisted of 4-6 sessions of compliance therapy, as described above, lasting 20-60 minutes roughly twice a week. The control treatment consisted of a similar number of sessions (mean 4.9) of supportive counseling in which the same therapists listened to the patients' concerns but declined to discuss treatment. Most of the therapy was carried out on the ward by a research psychiatrist (RK), with additional help from a clinical psychologist (PH).

In the first two sessions of compliance therapy patients were invited to review their history of illness and conceptualise the problem. In the next two sessions discussion became more specific, focusing on symptoms and the side effects of treatment. The benefits and drawbacks of drug treatment were considered, the patient's ambivalence was explored, and the therapist highlighted discrepancy between the patient's actions and beliefs, focusing on adaptive behaviours. In the last two sessions the stigma of drug treatment was tackled by considering that drugs are a freely chosen strategy to enhance the quality of life. Self-efficacy was encouraged and the value of staying well and thus the need for prophylactic or maintenance treatment was emphasised. The therapist encouraged the use of metaphors such as "protective layer" and "insurance policy." After discharge from hospital, all subjects received routine aftercare as determined by the clinical teams responsible for their care.

**Kemp 1998**

Four to six sessions of compliance therapy were given, divided into the following three

phases.

Phase I

The patient's illness history was reviewed, to ascertain his or her conceptualisation of

the illness and stance towards treatment. Where applicable an attempt was made to link medication cessation with relapse. Negative treatment experiences were acknowledged. Denial of illness or need for treatment was met with gentle enquiry into the ensuing social consequences or lifestyle disruption.

Phase 2

Ambivalence towards treatment was explored further. The therapist openly predicted certain common misgivings about treatment, such as fears of addiction, loss of control, loss of personality. Sometimes patients confuse symptoms and side-effects and misconceptions can be corrected. The natural tendency to stop medication when one feels well was discussed, and the meaning attached to medication was explored, that is identity as a 'sick person'. The patient was invited to weigh up the benefits and drawbacks of treatment, and the therapist 'homed in' on the benefits, especially when they emerged spontaneously. Symptoms reported by the patient were fed back as symptoms for treatment. Indirect benefits of medication were highlighted (e.g. getting on better with people). Metaphors were used, such as medication as a 'protective layer'. The therapist aimed to create a degree of cognitive dissonance in the patient, that is that poor compliance is actually disadvantageous for that individual in terms of his or her needs, lifestyle and goals.

Phase 3

Normalising rationales were used to deal with stigma. Analogies with physical illness requiring maintenance treatment were suggested, and the prevalence of illness was highlighted with examples of famous sufferers. The use of medication is reframed as a freely chosen strategy to enhance quality of life. The metaphor of medication as an 'insurance policy' to stay well was used. The therapist stressed the importance of staying well in order to reach certain self-identified goals and maintaining valued sources of fulfillment. The consequences of stopping medication were predicted, and characteristic prodromal symptoms identified, when early intervention could prevent a full-blown episode.

The therapy was given principally by R.K., with a minority seen by P.H. The therapists were trained in cognitive-behavioural psychotherapy, and attended a workshop on motivational interviewing. Regular supervision was undertaken to ensure consistency with regard to the delivery of the intervention.

**Laporte 2003**

Patients randomised to the standard education group with no particular emphasis on the necessity of strict compliance or specific information about the causes of anticoagulation instability, whereas these points were fully explained to the patients randomised to the intensive education group. Indeed, patients randomised to the intensive education group received information by visual material, were visited daily by nurses and physicians to repeat some items, and were tested daily about their education. The education, either standard or intensive, was given until hospital discharge.

**Lee 2006**

Pre-trial meet for all participants with clinical pharmacists involved individualized medication education (using standardized scripts), medications dispensed using an adherence aid (blister packs). Individualised educational interventions were performed to teach participants their drug names, indications, strengths, adverse effects and usage instructions.

Patients randomized to the pharmacy care group continued to meet with clinical pharmacists every 2 months, as previously performed in phase 1 of the study, and were provided blister -packed medications and also medication education as needed.

The blister multi-dose adherence package enables clear packaging and labeling of multiple medications in a disposable, punch card format. The translucent blister facilities visual verification of the card content. This medication packaging organizes the patient’s pills according to the daily dosing time and prevents them from working with multiple mediation bottles. Patients received combinations of morning, noon, evening or bedtime blister packs according to their regimen. Patients took the numbered blister that matched the day of the month.

**Levy 2000**

The intervention group was invited to attend a 1 hr consultation with one of the nurses beginning 2 weeks after entry to the study, followed by two more lasting half an hour, at 6-weekly intervals. The second and third could be substituted by a telephone call. Patients were phoned, by the nurse before each appointment in order to improve attendance rates. Patient's asthma control and management were assessed followed by education on recognition and self-treatment of episodes of asthma. Thus patients were taught to step-up medication when they recognized uncontrolled asthma using tests or symptoms. The advice was in accordance with national guidelines. Prescriptions were obtained from one of the doctors in the clinic or by providing the patient with a letter to their general practitioner. Patients presenting with severe asthma were referred immediately to the consultant.

The nurses:

(i) established the patients' understanding of asthma, their current treatment, their trigger factors and symptoms as well as guided self-management using PEF measurements;

(ii) assessed PEF, reversibility and inhaler technique (8);

(iii) expanded the patients knowledge to include a basic understanding of asthma and the medication used for prevention and relief; and

(iv) provided a validated, guided self-management credit card plan' (9,10) modified with lines drawn on the peak flow charts at 80%, 60% and 40% of best or predicted peak expiratory flow (PEF).

**Marquez 2004**

The intervention is a telephone call from a monitor, which had two objectives:

1. Establish the percentage compliance, decide whether the patient is a good or bad complier and, by means of this classification, make recommendations.  The caller ascertains the name, dose and dosing schedule of the lipid lowering drug taken, the number of tablets the patients has in their possession and the expiry date of the medication.  Participants are then told whether they are good or poor compliers based on the number of tablets they have left. The good compliers are congratulated and urged to continue their compliance [three statements were communicated to patients a) congratulations, you’re a great complier; b) thanks to your compliance your risk of suffering a heart attack will reduce significantly; and c) go on with your good compliance].  In the case of poor compliance, patients are urged to do better while explaining to them the benefits of good compliance [three statements were derived for these non-compliant patients a) you’ve stopped taking your tablets; b) to reduce your risk of heart disease you need to take the tablets every day; and c) we are encouraging you now so you can do so in future.]

2. Provide a reminder of the next appointment.

The intervention took place 7-10 days after the inclusion visit as well as 2 and 4 months later.

**Marquez 2006**

The groups that were created were: a control group (CG): patients receiving the intervention usually applied by their general practitioners; and an intervention group (IG): patients who, apart from a controlled intervention, also received an automatic monitor for blood pressure. The patients received a kit in their home containing the monitor, an instruction manual, a summary of the functions, and a card on which to note the measurements. They were advised to follow the HBPM programme, which consisted of measuring the BP 3 days a week (Tuesdays, Thursdays and Saturdays), twice before breakfast (0800 – 1000 h) and twice before supper (2000 – 2200 h) and record these results on the card (four times a day). The patients received a phone call to explain how to use the monitor and follow the programme.

**Peveler 1999**

The information leaflet was developed according to published principles and European Union directives. It contained information about the drug, unwanted effects, and what to do in the event of missing a dose. Patients were given drug counseling by a nurse at weeks 2 and 8, according to a written protocol. Four nurses acted as therapists: all had general nursing qualifications, had worked for at least 10 years since qualification, and had primary care experience; none had specialist mental health experience or training. Training for antidepressant drug counseling took 4 hours. Sessions included assessment of daily routine and lifestyle, attitudes to treatment, and understanding of the reasons for treatment. Education was given about depressive illness and related problems, self-help, and local resources. The importance of drug treatment was emphasised, and side effects and their management discussed. Advice was given about the use of reminders and cues, the need to continue treatment for up to 6 months, and what to do in the event of forgetting a dose. The feasibility of involving family or friends with medicine taking was explored.

**Piette 2000**

The core of the intervention was a series of automated telephone assessments designed to identify patients with health and self-care problems. These assessments were used to focus the efforts of a diabetes nurse educator on patients experiencing the greatest problems. The automated telephone calls also were used to deliver targeted and tailored self-care education messages.

Automated telephone calls. The automated calling component of the intervention was developed and implemented using a Teleminder Model IV automated telephone messaging computer (Decision Systems, Los Altos, California). The calls consisted of hierarchically structured messages composed of statements and queries recorded in a human voice. All calls were outbound (i.e. to the patients) and were placed at the times patients indicated were most convenient for them. Using up to six attempted calls, we sought to determine patients’ health status biweekly with a 5- to 8-minute assessment. Patients interacted with the system using their touch-tone keypad; responses were stored and determined the subsequent content of the message. Each query included a check for invalid responses and a prompt instructing patients to correct out-of-range values. Intervention patients received no training other than the general description provided as part of the informed consent process and a one- page summary provided in their enrollment packets. During each assessment, patients reported information about self-monitored blood glucose readings, self-

care, perceived glycemic control, and symptoms of poor glycemic control, foot problems, chest pain, and breathing problems. Assessments periodically included additional questions addressing issues unlikely to change on a biweekly basis (e.g., whether the patient had a retinal examination in the prior year). At the end of each assessment, patients were given the option to listen to a randomly cycling diabetes “health tip.” Health tips were 30 to 60 seconds in length and were based on literature published by the Centers for Disease Control and Prevention and the American Diabetes Association. Patients also were given the option to participate in a 3- to 5-minute interactive self-care education module focusing on diet and weight control.

After participating in the intervention for several months, patients were offered additional automated self- care education calls that focused on glucose self-monitoring, foot care, and medication adherence. Within these calls, patients reported speciﬁc barriers to self-care (e.g. no glucose monitoring strips) and received tailored education and advice. Within the medication adherence segment of the calls, patients were asked about their adherence to insulin, oral hypoglycemic medications, antihypertensive medications, and antilipidemic medications. For each type of medication, patients without adherence problems received positive feedback and reinforcement. Patients reporting less than optimal adherence were asked about speciﬁc barriers and were given advice about overcoming each barrier.

Telephone nurse follow-up. Each week, the automated assessment system generated reports organized according to the urgency of reported problems, and the nurse used these reports to prioritize patient contacts. During follow-up calls, the nurse addressed problems reported during the assessments and provided more general self- care education. The nurse was located outside of the clinics and had neither face-to-face contact with patients nor ready access to their medical records. She had access only

to medical record data that were abstracted at enrollment, automated assessment reports, and her notes from prior telephone contacts.

The nurse also made periodic calls to follow up on issues discussed in a prior week or check on patients who rarely responded to the automated calls. A small number

of contacts was initiated by patients using a toll-free telephone number. Depending on the nature and acuity of patients’ problems, the nurse contacted their primary

care physician via fax, voicemail, or pager.

**Remien 2005**

Grounded in Ewart’s social action theory, the brief intervention aimed to improve patients’ adherence to HIV/AIDS medical care regimens by fostering the support of their partners; in addition the intervention sought to help couples to address issues of sex and intimacy. The intervention was individually administered to each couple by a nurse practitioner through four 45 – 60 min sessions held over 5 weeks. The session content included structured discussions and instruction, as well as speciﬁc problem-solving and couple-communication exercises. Key components included education about the importance of adherence to avoid viral resistance and maintain health, identifying patterns of non-adherence, developing communication and problem-solving strategies to overcome adherence barriers, optimizing partner support, and building conﬁdence in the couple for achieving and maintaining improved adherence. Participants were reimbursed $20 per partner for each intervention session.

**Schroeder 2005**

Practice nurses invited participating individuals to attend, in addition to usual care, an adherence support session lasting a maximum of 20min, followed by a shorter reinforcement session (10min) two months later. The aim of the intervention was to provide an opportunity for patients to talk about any problems with their blood pressure lowering medication in a safe, non-threatening atmosphere, largely led by the patients themselves. During the consultation, practice nurses investigated whether patients under-stood their diagnosis and agreed with the treatment process. We encouraged practice nurses to address patient concerns with their medication and to agree tailored strategies to resolve any medication problems. We developed this pragmatic intervention using the self-regulatory model of illness behaviour. This takes into account the individual’s perception of symptoms, emotional responses to a health threat and coping strategies such as avoidance. The intervention was designed so that it could easily be introduced into routine practice. The only adherence-related training provided for the practice nurses was an explanation of issues around medication adherence by KS, lasting between 20–30min. We encouraged the nurses to find individual solutions to patients’ problems, taking into account their experience and knowledge of their patients.

BOX1 Common problem categories leading to difficulties adhering to a treatment regimen

Side effects

Size or taste of tablets

Number of doses a day

Non-acceptability of taking tablets

Forgetfulness

Non-comprehension

Total number of different tablets.

**Van Es 2001**

Participants in the experimental group received the same usual care from a pediatrician every 4 months. However, during these visits the pediatrician also discussed an asthma management zone system [3] with the participants. This system has been developed to instruct patients about disease characteristics, triggers for airway obstruction and treatment objectives. The pediatricians also discussed the peak expiratory flow measurements, which the participants had registered during the 2 weeks preceding the visit to the pediatrician. Furthermore, the four visits to the pediatrician were each combined with a visit to an asthma nurse. These experienced asthma nurses were specifically trained to apply their part of the intervention programme. The asthma nurses discussed several aspects of the disease individually with the participants, making use of drawings and written information based on. This written information was also intended to be read by the parents of the participants at home. For instance, the asthma nurse provided additional information on asthma medication and pulmonary conditions, and instructed patients how to use their inhaler. At each visit, the asthma nurse started by checking whether the participant still remembered and understood the information given during the preceding visit. The participants were encouraged to ask questions and to indicate what they wanted to know about having asthma. Every participant also attended three group sessions (four to eight participants per group). These group sessions took place once a week, after the three individual sessions with the asthma nurse had taken place. During the group sessions, participants discussed how they coped with their asthma and role-played several difficult situations under the supervision of the same asthma nurse. The discussions included the following issues: communication with a doctor, talking with peers about having asthma, attitude towards asthma and asthma medication and refusing to accept a cigarette. To support this discussion, the participants were shown a video film, which was purpose-made for the group sessions and showed several situations in which having asthma can be difficult for adolescents. After the three group sessions had been completed, a fourth individual visit to the asthma nurse took place. The objective of this final visit was to review all aspects of the preceding visits to the asthma nurse. The participants also received a written summary of the group sessions they had attended. Each session with the asthma nurse last approximately 30 minutes and each group session 90.

**Vergowen 2005**

Prior to every scheduled visit (7 visits in 26 weeks), the patients received a newsletter by mail. These letters reviewed the biology and symptoms, as well as risk factors for depression. The importance of antidepressant medication was emphasized, and its eﬀects and side-eﬀects were discussed. In addition, the need to continue treatment for up to 6 months was explained. Patients were also provided vignettes of depressive patients successfully treated with anti- depressant medication. The social stigma of depressive patients, the misconceptions about the potential to become dependent of antidepressant medication, and the false belief that depression is a sign of weakness were challenged. The importance of social support was addressed. Patients were instructed to discuss the topics of the newsletters with their GPs to increase their participation in the treatment. Patients were also asked to complete homework assignments: (1) to ﬁll out a questionnaire addressing the perceived costs and beneﬁts of treatment with antidepressant medication, (2) to plan activities, and (3) to discuss their illness and treatment with signiﬁcant others to enhance social support.

Prior to each visit, the GPs received a summary of the content of the newsletter and the homework assignments and were stimulated to discuss the topics and homework assignments with the patient. The GPs were asked to help patients clarify the potential beneﬁts of taking antidepressant medication and to challenge perceived costs of taking antidepressant medication. This instruction and the ﬁrst homework assignment were designed to build on general principles of motivational interviewing.

**Volume 2001**

Intervention reported in paper 1:Treatment pharmacies, for example, used an initial interview and frequent follow-up communication with the patient and other caregivers. In addition, pharmaceutical care interventions were often due to an in-depth review of the information collected by establishing a therapeutic relationship with the patient as opposed to being triggered by the receipt of a prescription, as was the case in the control pharmaicies.

Intervention reported in paper 2: The treatment pharmacists initiated pharmaceutical care by arranging 30- to 45-minute interviews with each of the research patients. The interviews were used to build a good working knowledge of the patients' health needs so that pharmacists could assess patients' drug-related needs. The Pharmacists' Management of Drug-Related Problems (PMDRP) form was used to acquire relevant patient data. In addition, to facilitate critical thinking and problem solving, the PMDRP form prompted the pharmacists with specific questions for each category of data collected. When pharmacists were not familiar with a specific disease state or its current treatment, they consulted the Therapeutic Thought Process worksheets.

**Walley 2001**

Family-member direct observation of treatment—i.e., supervision by a family member. The patient was assisted in the selection of a concerned and influential family member as supervisor. The family member was oriented on his or her role. The patient (or family member) collected drugs fortnightly from the health facility most convenient for him or her. The supervisor was taught how to record drug-taking using a specially designed form, and was made aware of the importance of observing drug-taking and of encouraging the patient to complete treatment. Drugs were delivered monthly to the health facilities, at which time the health workers’ work was supervised.

**Webber** **2004**

After randomization, study participants in the intervention arm received the address of one of the psychotherapists and were asked to schedule by themselves a first appointment. The allocation of participants to psychotherapists was at random, based on the availability of the psychotherapists who indicated the number of participants they would care for prior to the start of the study. The frequency of appointments with the psychotherapists was not predetermined by the study protocol but was worked out by participants and psychotherapists during the course of the study. The protocol defined a minimum of three and a maximum of 25 sessions within the 1-year study period. An intervention lasted 45 min on average.

The participants were informed that the study was not proposed because the physicians providing care felt that there was a psychological problem or a psychiatric disease to address but rather to support adherence to drug therapy using techniques of cognitive behaviour therapy, provided by specialists in this field. The psychotherapists were instructed to define at first visit, together with the participants, at least two goals for future interventions. At least one of the goals had to be related to medication adherence, but participants and psychotherapists were free to identify additional goals not related to anti-HIV therapy. The method of intervention had to be based on concepts of cognitive behaviour therapy.
