# Supplementary material for: Using qualitative evidence on patients’ views to help understand variation in effectiveness of complex interventions: a qualitative comparative analysis
Source: Trials. 2013 Jun 18;14:179. doi: 10.1186/1745-6215-14-179 (PMC3693880; doi:10.1186/1745-6215-14-179)
Supplement: Additional file 4: Table S1 — Representation of suggestions in effective trials and QCA solution displaying the six pathways to effectiveness. Table S2. Representation of suggestions in ineffective trials and QCA solution displaying the four pathways to ineffectiveness. [file 1745-6215-14-179-S4.doc]

**Table S1: Representation of suggestions ^1 ,2^ in effective trials and QCA solution^3^** displaying the six pathways to effectiveness

| Effective trialed intervention | Nine patient suggestions on how to promote adherence | | | | | | | | |
| --- | --- | --- | --- | --- | --- | --- | --- | --- | --- |
|  | A focus on personal risk factors | Enhance con-venience of taking drug | Information on side effects | Explore attitude to drug/disease | Emphasis on the value of adherence | Clear/  appropriate  information | Discusses the incidence of missed drug | No symptoms does not mean stop taking drug | Focus on improve social support |
| Marquez 2004 [30] | - | - | - | - | ✓ | - | - | - | - |
| Marquez 2006 [31] | - | - | - | - | ✓ | - | - | - | - |
| Schaffer 2004 [30] | - | - | - | - | ✓ | ✓ C | - | - C | - C |
| Hill 2001 [24] | - | - | ✓ | - | - | ✓C | - | - C | - C |
| Lee 2006 [28] | - | ✓ | ✓ | - | - | ✓ C | - | - C | - C |
| Farber 2004 [22] | ✓ | - | - | ✓ | - | ✓ C | - | - C | - C |
| Berrien 2004 [21] | ✓ | ✓ | - | - | ✓ | ✓ | ✓ | - | ✓ |
| Piette 2000 [28] | ✓ | - | - | - | ✓ | - | ✓ | - | - |
| Haynes 1976 [23] | ✓ | - | - | - | - | - | ✓ | - | - |
| Kemp 1996 [25] | ✓ | - | ✓ | ✓ | ✓ | - | - | ✓ | - |
| Kemp 1998 [26] | ✓ | - | ✓ | ✓ | ✓ | - | - | ✓ | - |
| Levy 2000 [29] | ✓ | - | - | - | ✓ | ✓ | - | ✓ | - |
| Peveler 1999 [32] | ✓ | ✓ | ✓ | - | ✓ | - | - | ✓ | ✓ |

1. ✓ = the intervention contains a component that matches the suggestion. 2. - = the intervention does not contain a component that matches the suggestion. 3. Parsimonious solution.

Pathway One. Present: ‘A focus on personal risk factors’.

|  |
| --- |

Pathway Two. Present: ‘Emphasis on the value of adherence’. Absent: ‘Discuss the incidence of missed drugs’ and ‘A focus on improving social support’;

|  |
| --- |

Pathway Three. Present: ‘Clear/appropriate information on taking drug. Absent: ‘Explore attitudes to drug/disease’ and ‘No symptoms does not mean stop taking drug’;

|  |
| --- |

Pathway Four. Present: ‘Clear/appropriate information on taking drug’. Absent: ‘Discus the incidence of missed drugs’ and ‘No symptoms does not mean stop taking drug;

|  |
| --- |

Pathway Five. Present: ‘Emphasis on the value of adherence’. Absent: ‘No symptoms does not mean stop taking drug’ and ‘A focus on improving social support’;

|  |
| --- |

Pathway Six. Present: ‘Clear/appropriate information on taking drug’. Absent: ‘No symptoms does not mean stop taking drug n’ and ‘A focus on improving social support’.

| C |
| --- |

**Table S2: Representation of suggestions^1, 2^ in ineffective trials and QCA solution^3^ displaying the four pathways to ineffectiveness**

| Trialed intervention | Nine patient suggestions on how to promote adherence | | | | | | | | |
| --- | --- | --- | --- | --- | --- | --- | --- | --- | --- |
|  | A focus on personal risk factors | Enhance convenience of taking drug | Information on side effects | Explores attitude to drug/condition | Emphasis on the value of adherence | Clear or  appropriate  information | Discusses incidence of missed drug | No symptoms does not mean stopping drug | Focus on improve social support |
| Laporte 2003 [27] | - | - | - | - | ✓ | ✓ | - | ✓ | - |
| Vergowen 2005 [38] | - | - | ✓ | ✓ | ✓ | - | ✓ | ✓ | ✓ |
| Remien 2005 [34] | - | - | - | - | ✓ | - | ✓ | - | ✓ |
| Van Es 2001 [32] | - | - | - | ✓ | - | ✓ | ✓ | - | ✓ |
| Volume 2001 [39] | - | - | - | - | - | - | - | - | - |
| Walley 2001 [40] | - | - | - | - | - | - | - | - | ✓ |
| Webber 2004 [41] | - | - | - | - | - | - | - | - | - |
| Schroeder 2005 [36] | - | ✓ | ✓ | ✓ | - | - | ✓ | - | - |

1. ✓ = the intervention contains a component that matches the suggestion. 2. - = the intervention does not contain a component that matches the suggestion. 3. Parsimonious solution.

Pathway One: Present ‘Discusses incidence of missed drugs’, Absent: ‘A focus on personal risk factors’;

|  |
| --- |

Pathway two: Absent: ‘Provides information on side effects’ and ‘Emphasis on the value of adherence’ or ‘A focus on personal risk factors’;

|  |
| --- |

Pathway three: Present ‘No symptoms does not mean stopping medication,’ Absent ‘A focus on personal risk factors’;

|  |
| --- |

Pathway four: Absent: ‘A focus on personal risk factors’ and ‘Emphasis on the value of adherence’ and ‘Clear information on taking drug’.

| - |
| --- |
